# Factors Associated with the Severity of Clinical Mastitis

**DOI:** 10.3390/pathogens11101089

**Published:** 2022-09-24

**Authors:** Frederik Fredebeul-Krein, Anne Schmenger, Nicole Wente, Yanchao Zhang, Volker Krömker

**Affiliations:** 1 Department of Microbiology, Faculty of Mechanical and Bioprocess Engineering, University of Applied Sciences and Arts, 30453 Hannover, Germany; 2 Department of Veterinary and Animal Sciences, University of Copenhagen, 1870 Frederiksberg, Denmark

**Keywords:** bovine mastitis, severity score, risk factors, severe mastitis

## Abstract

Severe mastitis can lead to considerable disturbances in the cows’ general condition and even to septicemia and death. The aim of this cross-sectional study was to identify factors associated with the severity of the clinical expression of mastitis. *Streptococcus* (*Str.*) *uberis* (29.9%) was the most frequently isolated pathogen, followed by coliform bacteria (22.3%). The majority of all mastitis cases (n = 854) in this study were either mild or moderate, but 21.1% were severe. It can be deduced that the combination of coliform pathogens and increasing pathogen shedding of these showed associations with severe mastitis. Furthermore, animal-related factors associated with severe disease progression were stages of lactation, and previous diseases in the period prior to the mastitis episode. Cows in early lactation had more severe mastitis. Ketosis and uterine diseases in temporal relation to the mastitis were associated with more severe mastitis in the diseased cows. Hypocalcemia was significantly associated with milder mastitis. As another factor, treatment with corticosteroids within two weeks before mastitis was associated with higher severity of mastitis. Knowledge of these risk factors may provide the basis for randomized controlled trials of the exact influence of these on the severity of mastitis.

## 1. Introduction

Mastitis is one of the most commonly occurring diseases on dairy farms. Severe mastitis is a decisive issue on dairy farms for animal welfare, animal protection, and profitability reasons [1]. Research on mastitis has not adequately investigated the factors influencing the severity of clinical expression of mastitis. The classification of severity is based on the definitions of the International Dairy Federation [2]. Cases are classified as MS 1 (mild clinical mastitis) if only deviations in the nature of the milk (color, consistency, and viscosity) appear. If local inflammatory symptoms (swelling, induration, painfulness, redness, and increased warmth) are also present, cases are classified as MS 2 (moderate clinical mastitis). MS 3 (severe clinical mastitis) is characterized by complementary general clinical signs of illness (fever, hypothermia, recumbency, and aversion to feed) [2,3]. In previous studies, the distribution of mild, moderate, and severe cases varied between 48–55%, 36–37%, and 9–15% [3,4]. In the study by Wenz et al., 2001, 35% of cows with clinical mastitis caused by *Escherichia* (*E.*) *coli* or *Klebsiella* (*K*.) *pneumoniae* died during the study [5]. Moreover, in the study by Wenz et al., 2001, 32% of cows with severe coliform mastitis developed bacteremia [5]. Microbiological and clinical cure of mastitis are associated with the severity of mastitis, with lower cure rates reflecting an association with more severe mastitis [6,7]. Thus, as the severity increases, the prognosis for the diseased quarter and survival rates of the infected individuals decrease [5,8,9,10]. Severe clinical mastitis is also associated with irreparable tissue damage in the diseased glandular quarter if caused by *E. coli* [8] and can lead to septicemia and death [5,8,9,10].

Mastitis is multifactorial in its course and clinical presentation. Factors influencing its severity are attributed to the pathogen and the animal, among other factors [9,11]. In the last decades, some studies have focused on the relationship between the causative pathogen and mastitis severity. Thereby, most of the studies concentrated on the association between the pathogen *E. coli* and severe mastitis [4,12,13]. Among veterinarians and farmers, coli mastitis is sometimes used as a synonym for severe clinical mastitis [12]. Oliveira et al., 2013, showed in their study the distribution of pathogens among severe mastitis cases. They found that 75.3% of severe mastitis cases were caused by Gram-negative pathogens and that *E. coli* accounted for two-thirds of these [4]. However, recent evidence suggested that half of the severe clinical mastitis cases could be caused by Gram-positive pathogens, primarily *Streptococcus* (*Str.*) *uberis* [3]. Both Gram-positive and Gram-negative bacteria have been shown to have numerous virulence factors, and associations between these virulence factors and severe mastitis have been investigated in some studies [14,15,16,17]. The unanimous conclusion of these studies was that the virulence factors of the pathogens had no significant effect on the severity of mastitis [14,15,16,17].

According to Burvenich et al., 2003 [12], and Wenz et al., 2006 [15], animal-specific factors influence the severity more than pathogen factors of *E. coli*. The immune system of the dairy cow significantly influences the incidence of mastitis and the severity of its clinical manifestations, and it has been shown that severe mastitis occurs more frequently in early lactation [18]. Immunoglobulin G and the number and function of polymorphonuclear neutrophil granulocytes (PMN) are parts of the innate immune system [12,19,20]. Furthermore, in early lactation, metabolic and hormonal changes lead to an immunosuppression and have a negative impact on the number and function of PMN [12,21]. Metabolic immunosuppression with low stress tolerances can be triggered in the puerperium by negative energy balances (NEB) [22]. Many dairy cows experience an NEB during the postpartum period because the feed intake capacity at this time cannot meet the energy requirements for increasing milk production and uterine regeneration. Predisposing factors for NEB include overconditioning and very high milk yields [12]. Retentio secundinarum may also negatively affect the number and function of PMNs in the puerperium through lower levels of interleukin-8 [23]. Schukken et al. (1988) noted that retentio secundinarum was associated with an increased risk of severe mastitis [24]. The decreased number and function of PMNs affect bacterial numbers in the mammary gland and thus, the clinical expression of mastitis is negative due to inadequate intervention in bacterial growth and elimination [12,14]. For the pathogen *E. coli*, the bacteria count in the mammary gland at the onset of mastitis shows an association with the severity such that higher pathogen numbers are associated with more severe mastitis [11]. More severe clinical manifestations of mastitis depend on either a release of excessive amounts of endotoxins or a stronger host response [15]. Endotoxins are a decay product of the outer cell membrane of Gram-negative bacteria, such as *E. coli* [25]. When released, endotoxins stimulate the immune system to activate endogenous inflammatory mediators at the cellular level, such as the endotoxin-triggered cytokine tumor necrosis factor alpha (TNF-α) [25]. Immunosuppressions are also often associated with oxidative stress and low antioxidant defense during early lactation [26]. Deficient supplies of the antioxidants vitamin E or selenium negatively affect the severity of mastitis, as these antioxidants originally protect PMN from the destructive nature of toxic oxygen molecules [27,28]. In dairy cows, vitamin E deficiency is mainly evident in the peripartum period [28]. Several studies have shown that primiparous cows are less likely to develop severe mastitis than multiparous dairy cows [12,18,29,30]; however, this association is not always seen [4]. The difference in risk of severe mastitis, between primiparous and multiparous cows, can partly be due to primiparous cows having a higher viability of PMN [12,18,29,30] and higher production of neutrophil reactive oxygen species (ROS) than multiparous cows [12]. Vaccinations against the mastitis-associated microorganisms *Staphylococcus* (*S*.) *aureus*, *Str. uberis*, and *E. coli* have resulted in milder courses of mastitis, but the protection against new infections by inhibiting bacterial growth due to elevated IgG1 and IgG 2 is limited [14,31,32,33]. If an animal suffers from multiple cases of mastitis caused by *E. coli*, the initial infection with *E. coli* is often more severe than the subsequent infections [34]. However, Oliveira et al., 2013 were unable to demonstrate any influence from previously occurring episodes of mastitis on the severity of subsequent cases in the current lactation [4]. This indicates that there are different views in the scientific community on how the severity of mastitis is influenced by prior cases of mastitis. One study reported an association between severe mastitis cases and high daily milk production in the dairy herd improvement (DHI) test before enrollment of clinical mastitis [4]. The daily milk production in the DHI test after enrollment of clinical mastitis showed no differences between the severity scores [4]. The deviation in daily milk production between the DHI test before and the DHI test after enrolling the mastitis cases was greatest for cows with severe mastitis [4]. In one study, no association was shown between the somatic cell count from the diseased cows at the onset of mastitis and severity [25].

The development of mastitis depends on exposure to microorganisms, the cows’ local and systemic defenses, and environmentally associated risk factors [35]. Severe mastitis can vary in clinical appearance on a cow-by-cow basis [36]. The type and number of microorganisms, in addition to the cows’ immune defense, lead to a variation in symptoms [10]. The severity of clinical symptoms is influenced by an interplay of predisposing factors, endogenous defense mechanisms, functionality of glandular tissue in the udder, and treatment success [37,38]. There is still a lack of evidence of factors influencing the severity of clinical expression. As it is unclear which traits characterize animals under risk for developing severe mastitis, the management of high-risk animals should be facilitated by identifying starting points in association with severe mastitis. Further knowledge of risk factors can form the basis for randomized controlled trials focusing on the exact influence of these on the severity of mastitis. Thus, the aim of the present cross-sectional field study was to determine factors associated with severe mastitis.

## 2. Results

### 2.1. Descriptive Results

A total of 854 udder quarter cases of clinical mastitis were enrolled in this study. The majority of the mastitis cases in this study were either mild or moderate, but 21.1% were severe (Table 1). A large proportion of mastitis cases were caused by environmentally associated microorganisms. The distribution of bacteriological findings is presented in Table 1, showing that the most frequently isolated pathogen was *Str. uberis* (29.9%), followed by coliform pathogens (22.2%), and no growth (20.3%). Coliform pathogens were the most common finding (42.2%) at severe cases of mastitis, followed by *Str. uberis* (23.3%), and no growth (21.3%). Moderate mastitis cases were mostly caused by *Str. uberis* (39.9%), followed by coliform microorganisms, and no growth (Table 1). In the mild cases, no growth was most common (31.7% of the cases), followed by *S. uberis*, and non-aureus staphylococci (NaS) and *Corynebacterium* species (Table 1). The pathogen *Str. uberis* was significantly more common in udder quarters with moderate mastitis than in udder quarters with mild and severe cases of mastitis. There was no significant difference in the findings of *Str. uberis* between mild or severe cases (*p* = 0.651). Moreover, the findings of coliform pathogens were significantly more common in moderate and severe cases compared to mild ones (*p* < 0.001), and in severe compared to moderate cases (*p* < 0.001).

Most clinical mastitis cases in this study occurred in the first 100 days of lactation (44.0%). Clinical mastitis occurred in equal proportions between the 101st and 200th days of lactation (27.3%), and in late lactation after the 200th day of lactation (28.7%). Severe cases were significantly more common in early lactation than in mid- and late lactation (Table 2).

A previous illness in the 14 days before the onset of mastitis was recorded in 12.5% of cases in this study. Among all mastitis cases in the study, previous diseases in total were significantly more common in cows with severe mastitis than in cows with mild or moderate mastitis. Ketosis (yes/no) and uterine diseases (yes/no) were significantly more common in severe mastitis cases than in moderate and mild mastitis cases. Mastitis and hypocalcemia occurred more frequently as a previous disease in mild mastitis cases than in moderate and severe mastitis cases (Table 2).

Of the 854 cows participating in this study, 2.7% were treated with corticosteroids within two weeks prior to mastitis, and pretreatment with corticosteroids was significantly more common in severe cases than in moderate and mild cases.

From a total of the 854 cows included in the study, 61.3% (524 cases) were in parity three or higher, 21.8% (186 cases) were in parity two, and 16.9% (144 cases) were in first parity (Table 2).

In measurements of pathogen shedding, high pathogen shedding above 5000 colony-forming units per milliliter was significantly more common among severe mastitis cases than among moderate and mild mastitis cases. Severe cases were significantly more common in cows with a high (>40 kg milk) daily milk production in the last DHI before the mastitis than in cows with moderate or mild cases. Dairy cows with a milk fat content below 4% in the last DHI before mastitis had a significantly higher incidence in the severe cases than in the moderate and mild cases. Lameness in cows occurred less in association with mild mastitis than in association with moderate and severe mastitis. In 25.5% of the cases in this study, the cows had already contracted mastitis in the current lactation. Further mastitis in the current lactation was recorded more commonly in mild cases than in moderate and severe cases. Scarce body condition scores (≤2.5) in the diseased animal at the time of mastitis were more common in severe mastitis than in non-severe mastitis. Severe mastitis emerged significantly more commonly with fever (≥39.5) and not maintained feed intake behavior than mild and moderate mastitis cases. The udder quarters were affected in equal proportion independent of position and severity. Dairy cows vaccinated in the 14 days prior to mastitis had a higher incidence in mild mastitis than in moderate and severe mastitis cases. The distribution of severity with respect to seasons showed that severe mastitis was significantly more frequent in summer compared to moderate and mild mastitis cases.

### 2.2. Results of Final Generalized Linear Mixed Models with Binominal Response

Associations between the severity score of occurring clinical mastitis and risk factors were examined with generalized linear mixed models and binomial response (severe and non-severe (logistic regression)) after pre-screening for variable selection in univariable analysis. The mastitis-causing pathogen group was not associated with the clinical expression of severity of mastitis (*p* = 0.223 in the final model, not shown in the table), but it was still a univariable significant factor. The same applies to the ordinal variable “pathogen shedding” (*p* = 0.172 in the final model, not shown in the table). The interaction between pathogen group and pathogen shedding was significantly associated with the severity of clinical expression of mastitis (*p* < 0.001) (Table 3). On comparing the pathogen groups with one another, the combination of the coliform pathogens and increasing pathogen shedding (>5000 cfu/mL) (OR 1.63, CI 1.34–1.99), and the other pathogens and increasing pathogen shedding (OR 1.37, CI 1.03–1.83) were associated with more severe courses of mastitis. Other pathogens included Prototheca species, Bacillus species, Enterococcus species, yeast, Pseudomonas species, Str. dysgalactiae, Str. agalactiae, other streptococci, Trueperella (T.) pyogenes, and Serratia species. The stage of lactation was associated with the severity of clinical expression of mastitis (*p* = 0.002) (Table 3). Cows in early lactation had higher odds (OR 2.59, CI 1.51–4.44) of having a severe mastitis than cows in late lactation. Having a disease within 14 days before the onset of mastitis was associated with the severity of clinical expression of mastitis (*p* = 0.023) (Table 3). This significant association was mainly due to hypocalcemia cases. Cows with hypocalcemia prior to the mastitis case had lower odds (OR 0.10, CI 0.02–0.67) of having a severe mastitis case than cows without disease prior to the mastitis case. Corticosteroids used within 14 days prior to the onset of mastitis influenced the severity of clinical expression of mastitis (*p* = 0.003) (Table 3). Cows that had been treated with corticosteroids had higher odds (OR 6.11, CI 1.834–20.33) of having a severe case of mastitis than cows that had not been treated with corticosteroids.

## 3. Discussion

The aim of the study was to identify factors associated with the severity of the clinical expression of mastitis. If animals treated with corticosteroids developed mastitis within the following 14 days, this was associated with a more severe course in relation of the reference category of no treatment. We have two theories to explain this. One is that cortisone-based agents lead to immunosuppression. This suppression of the immune system may also result in a reduction in the number and function of PMN. According to Roth et al., 1982, glucocorticoids lead to reduced neutrophil granulocyte margination. This stage of diapedesis influences the flooding of leukocytes in the tissue [39]. The disease of origin and the indication of corticosteroid therapy could affect the course of mastitis. In early lactation, ketosis was one of the most common indications of corticosteroid therapy. On reviewing the multicollinearity between ketosis as a previous disease and the treatment of corticosteroids in relation to the severity of clinical expression of mastitis, a relationship was evident. However, there must be other explanations besides ketosis. Further research is still needed to substantiate these findings.

Previous infection with diseases within 14 days prior to mastitis was associated with the severity of clinical expression. Dairy cows that had a history of hypocalcemia within two weeks prior to mastitis were significantly associated with non-severe mastitis courses in relation to the reference category of no diseases. As early as 1989, a study found that all cows exhibited severe loss of immune cell function in the weeks around parturition [40]. This immunosuppression was not enhanced by hypocalcemia. Increased incidence risk in hypocalcemic cows compared with nonhypocalcemic cows has been associated with lower teat end sphincter muscle tone and increased exposure of the teat to environmentally associated pathogens due to prolonged recumbency [40]. Combined therapy of hypocalcemia with rapid compensation of blood calcium levels, application of an anti-inflammatory agent, and fluid therapy may have an influence on milder severity of mastitis. In addition, cows that have had hypocalcemia may be under closer observation due to the known increased incidence risk, so mastitis is detected earlier. No association could be shown between mastitis as a previous disease and the severity of subsequent cases of mastitis, which was consistent with a previous study [4]. In another study using *E. coli* as an example, a reduction in the severity of secondary infections of *E. coli* was striking [34].

The majority of clinical mastitis cases in this study occurred in the first 100 days of lactation. Still, the stage of lactation was associated with severity of clinical expression of mastitis, where the odds of severe mastitis was higher in early lactation than in late lactation. It should be noted that the distribution of lactation stages was not balanced with respect to severity. Possible explanations lie in the weakening of the immune system due to metabolic and hormonal changes at the start of lactation. The metabolic disease ketosis mainly occurs in early lactation and influences the immune system [22,41,42]. Hormonal changes in the form of increased concentrations of sex steroids and glucocorticosteroids from the neuroendocrine system weaken the number and function of PMN around parturition and at the beginning of lactation [18]. Immunosuppression is often associated with oxidative stress and low antioxidant defense at this stage of lactation [26]. In another study, associations between severity, stage of lactation, and pathogen were investigated, and it was found that 70.3% of severe mastitis cases due to *Str. uberis* occurred in the first 100 days of lactation [43].

The combination of the pathogen group with increasing pathogen shedding was associated with the severity of clinical expression of mastitis. The interaction of coliform pathogens and increasing pathogen shedding was significantly associated with more severe mastitis. The interaction of other pathogens (not *Str. uberis*, coliform pathogens, no growth, NaS and *Corynebacterium* species, mixed infections, and *S. aureus*) with increasing pathogen shedding was also associated with more severe courses of mastitis. The combination of the pathogen group with increasing pathogen shedding thus seems to lead to significantly more severe mastitis. Regarding the coliform pathogens, this means that the presence of many coliform pathogens in milk was associated with more severe mastitis courses. In a previous study, higher pathogen counts for the pathogen *E. coli* were associated with more severe cases of mastitis [15]. Burvenich et al., 2003 also reported an association between a higher pathogen density of *E. coli* at the onset of mastitis with severe mastitis cases [12]. The extent of pathogen shedding in mastitis may be an indirect indicator of PMN number and function, and thus of pathogen elimination by PMN. In early lactation, this pathogen elimination is affected by PMN due to metabolic and hormonal changes [12]. In the presence of higher pathogen shedding of coliform pathogens, more severe clinical manifestations of mastitis may also depend on the excessive release of endotoxins, which are a decay product of the outer cell membrane of Gram-negative bacteria [15,25]. Therefore, high pathogen shedding of the aforementioned pathogen groups was considered a cause or a typical symptom of severe mastitis caused by coliform and other microorganisms. The mastitis-causing pathogen group was univariably significant, but no longer remained as the sole associated factor with the severity of clinical expression of mastitis in the last detailed analyses. The dominant microorganisms among severe mastitis cases were coliform pathogens, in that they were causative for 42.2% of all severe mastitis cases. An association of severe mastitis with coliform pathogens was shown in a study by Oliveira et al. in 2013 [4]. Bradley et al., 2001 [13], also showed that mastitis due to *Escherichia coli* was associated with more severe courses of mastitis and that this was most noticeable in early lactation. In the present study, the second most common cause of severe mastitis cases was *Str. uberis* with 23.3%. The order of microorganism as causative agents of severe mastitis was consistent in order with the results of Schmenger et al., 2020, such that severe mastitis was most commonly caused by coliform pathogens and second most commonly by *Str. uberis*. [3]. Severe mastitis cases were also primarily caused by environmentally associated pathogens. Cow-associated pathogens, such as *S. aureus* and ß-hemolytic streptococci, were commonly associated with subclinical and mild mastitis [44]. Similar courses were mostly shown by the group of minor pathogens to which the *non-aureus staphylococci* belong, for example [44]. It is possible that the mastitis-causing pathogen would remain a predominant factor relating to the severity of the mastitis at the end of the study if even more mastitis cases were sampled.

In this study, the severity was distributed among 35.8% mild mastitis cases, 43.1% moderate mastitis cases, and 21.1% severe mastitis cases. In comparison, we documented more severe mastitis cases in percentage than Schmenger et al., 2020 (9.1%) and Oliveira et al., 2013 (15.3%). The variation in the distribution of severity in the present study may be explained by the fact that probably not every farm recorded and sampled all mild mastitis cases in a year. This could be especially true in robot herds, where mild mastitis cases were often detected later or not at all. A recent study by Bausewein et al., 2022 was able to show that fewer mild and moderate mastitis cases are detected in automatic milking systems [45]. The pathogen *Str. uberis* was detected most frequently in the present study with a prevalence of 29.9%. The second most commonly occurring bacterial group was coliform pathogens, accounting for 22.2%. Most clinical mastitis cases were caused by environmentally associated microorganisms. These results are consistent with other studies [3,10]. According to Klaas et al., 2018 [46], there is a pre-ponderance of environmentally associated pathogens as causes of mastitis due to the fact that in recent decades, more and more farms have implemented mastitis prevention programs to reduce the highly contagious cow-associated pathogens by optimizing the milking process and dry cow management. This was also evident from the low incidence of the cow-associated pathogen *S. aureus* in 4.8% of all clinical mastitis cases. In 20.3% of cases, no bacterial growth was detectable. A common cause of no bacterial growth is an insufficient bacterial count, which mainly occurs in mild mastitis [47]. In their study, Krömker et al., 2010, were able to show a distribution of pathogens in mastitis by other examination methods, in which no growth could be detected by usual examination methods [47]. There, clinical mastitis without bacterial growth was caused by low bacteria numbers of *non-aureus staphylococci* in 27.5% of cases, *E. coli* and other coliform pathogens in 24.4% of cases, and *Corynebacterium* species in 6.7% of cases [47]. A total of 17.1% of milk samples without bacterial growth yielded inhibitory substances [47]. Detection of lipopolysaccharides suggested a self-limiting mechanism of Gram-negative pathogens in 3.6% of milk samples without bacterial growth [47].

The influence of vaccination against mastitis-associated microorganisms on the severity of the course of mastitis could not be adequately verified within the scope of this study because only one farm used such a vaccination. When discussing the methods used in the present study, a different farm selection would be conceivable by reducing the number of farms and increasing the farm size. In addition, pure random selection of herds could be performed to further reduce potential bias effects from more targeted selection. The selected dairy farms are localized in Northwestern Germany, especially in the districts of Hochsauerlandkreis, Soest, Paderborn, Märkischer Kreis, and Waldeck-Frankenberg. In a 2020 annual report from the *Landeskontrollverband Nordrhein-Westfalen e.V.*, the average farm size in South Westphalia was 87.5 cows per farm with an average 305-day yield of 9543 kg and an average bulk milk somatic cell count of 214 kcells per milliliter [48]. Further north and further west, there are also many dairy farms with similar structures, but here, farms are often larger. Furthermore, selection criteria on udder health of the farms could be added. This could, for example, increase the sampling rate of mild mastitis. Regarding the evaluation of the body condition score and lameness, a veterinary evaluation alone would possibly increase accuracy. In this study, no more detailed investigations were conducted at the cellular, immunological level. Knowledge of the risk factors of corticosteroid therapy within two weeks prior to mastitis and stage of lactation can provide the basis for randomized controlled trials of the exact influence of these two factors on the severity of mastitis. Furthermore, research gaps exist with regard to the extent of influence of herd-related factors on the severity of this disease.

## 4. Materials and Methods

All applicable guidelines for the care and use of animals were followed. The study was approved by the Animal Welfare Committee of the University of Veterinary Medicine Hannover, Foundation, Hannover, Germany (file reference: TVO-2020-V-63). An application for a license for animal testing was not required by the local government due to the study design. The study complied with the International Guiding Principles for Biomedical Research Involving Animals (1985).

### 4.1. Herds and Study Design

The cross-sectional study was conducted on 65 dairy farms in Northwestern Germany located in North Rhine-Westphalia and Hesse in the period between May 2020 and May 2021. The dairy farms for this study were selected to a large extent from the clientele of *Tierärztliche Gemeinschaftspraxis Büren FGS-GmbH*, Büren, Germany and supplemented by other motivated dairy farms from the region. Criteria for farms to participate in the study included conducting a monthly DHI test. Data from the last milk control before the onset of clinical mastitis were analyzed. Participating farms with herd sizes between 35 and 390 dairy cows of Holstein Friesian and Brown Swiss breeds had an average 305-day milk yield between 6000 and 12,700 kg. The average bulk milk somatic cell count varied between 100,000 and 470,000 cells per mL. In a 2020 annual report from the *Landeskontrollverband Nordrhein-Westfalen e.V.*, the average farm size in South Westphalia was 87.5 cows per farm with an average 305-day yield of 9543 kg and an average bulk milk somatic cell count of 214 cells per milliliter [48].

### 4.2. Sampling

Milk samples of clinical mastitis were collected in the study by the farm managers and veterinarians from the udder quarters with clinical signs. Milk samples were collected in test tubes containing the preserving agent boric acid (Ly20) [49] and were shipped directly to the laboratory. Farm managers and veterinarians were trained in clean milk sampling in accordance with Germany Veterinary Association guidelines [50] as well as brief assessment of animal-specific health parameters. The classification of clinical mastitis into severity levels was made based on International Dairy Federation definitions [2].

### 4.3. Protocol

Animal-specific health parameters were collected in a questionnaire at the time of sampling. The farm manager or veterinarian measured the rectal temperature and assigned this to hypothermia (below 38.0 °C), normal temperature (between 38.0 and 39.4 °C), or fever (above 39.4 °C). The localization of the diseased quarter and the individual feed intake behavior were recorded. It was reported whether the animal had already suffered from mastitis in the current lactation (yes/no). Further previous diseases (especially the most common puerperal diseases—yes/no), vaccinations (yes/no), treatments with corticosteroids (yes/no), and lameness (yes/no) over the period of 14 days before the occurrence of mastitis were recorded. In addition, the date of onset of mastitis was documented to test for seasonal predictors. At the end of the questionnaire, the farmer or veterinarian evaluated the body condition score (BCS) according to Edmonson et al. [51], modified according to Metzner et al. [52]. This was classified as underconditioned with a BCS less than/equal to 2.5, normal condition with a BCS between 2.75 and 3.5, or overconditioned with a BCS greater than/equal to 3.75.

### 4.4. Laboratory Procedures

Conventional cyto-microbiological diagnostic examinations were performed at the laboratory of Hannover University of Applied Sciences and Arts, Hannover, Germany in accordance with the guidelines of the German Veterinary Association [50], which are comparable to the recommendations of National Mastitis Council [53]. Then, 10 μL of each well-mixed milk sample was plated with a sterile calibrated loop on a quadrant of an aesculin blood agar plate (Thermo Fisher Scientific, Langenselbold, Germany). Plates were incubated for at least 48 h at 37 °C under aerobic conditions. Isolates were Gram stained to assist in organism identification. Furthermore, morphology of colonies, aesculin hydrolysis, catalase reactivity (3% H_2_O_2_; Merck, Darmstadt, Germany), and hemolysis patterns were used for identification. Gram-positive and catalase-positive cocci were identified as staphylococci. For differentiation of *S. aureus*, a clumping factor test was performed (Staph Plus Kit, DiaMondiaL, Vienna, Austria). Other staphylococci were referred to as *non-aureus staphylococci*. Gram-positive and catalase-negative cocci were identified as streptococci. For differentiation of aesculin hydrolyzing cocci, modified Rambach agar was used [54]. -d-Galactosidase-positive and aesculin hydrolyzing cocci were identified as *Str. uberis*. Aesculin hydrolyzing, -d-galactosidase-negative cocci were identified as enterococci. ß-hemolytic streptococci were characterized by Lancefield serotyping (DiaMondiaL Streptococcal Extraction Kit Sekisui, Virotech Diagnostics GmbH, Dietzenbach, Germany). Streptococci in group C were referred to as *Str. dysgalactiae*, in group B as *Str. agalactiae*. Gram-positive, ß-hemolytic, catalase-negative irregular rods with V- or Y-shaped configurations were identified as *T. pyogenes*. Gram-positive, catalase-positive, asporogenic colonies on aesculin blood agar were identified as coryneform bacteria. *Bacillus* species form colonies on aesculin blood agar which are catalase-positive and appear as Gram-positive rods which form endospores. Gram-negative and cytochrome oxidase negative (Bactident oxidase, Merck KGaA, Darmstadt, Germany) rods were further differentiated using Chromocult Coliform Agar (Merck KGaA). After incubation at 37 °C for 24 h, *E. coli* formed blue colonies and other coliforms, pink-red colonies. Gram-negative rods showing no mobility during the performance of the oxidative fermentative test were identified as *Klebsiella* species. Gram-negative, catalase-positive, and cytochrome oxidase-positive rod-shaped bacteria showing oxidative glucose degradation were identified as *Pseudomonas* species. Yeasts, molds, and *Prototheca* species were differentiated microscopically. Environment-associated, mastitis-causing microorganisms (*Str. uberis*, *E. coli*, *Klebsiella* species, coliform bacteria, yeasts, *Pseudomonas* species, and *Prototheca* species) and NaS were recorded as a microbiologically positive result if at least ≥5 cfu/0.01 mL were cultured. Based on the recommendations of the National Mastitis Council [53], samples with two identified pathogens were considered a mixed infection, whereas samples with more than two pathogens were described as contaminated, unless a colony of a cow-associated microorganism (*S. aureus*, *Str. agalactiae*, *Str. dysgalactiae* or *T. pyogenes*) was found [3]. In the context of this study, pathogen shedding was also documented. For this purpose, the number of colonies grown on the blood agar was counted semi-quantitatively. A distinction was therefore made between three levels of pathogen shedding. In stage 1, 1 to 10 colony-forming units (cfu) were grown in 0.01 mL, in stage 2, 11 to 50 cfu were grown in 0.01 mL, and in stage 3, more than 50 cfu were grown in 0.01 mL of blood agar.

### 4.5. Data of the Dairy Herd Improvement Tests

Dairy herd improvement test data were analyzed on an animal-specific basis. Enrolled data were days in milk (DIM) at the onset of mastitis, parity at the onset of mastitis, daily milk production in the last DHI before mastitis, and milk yield of 305-day pre-lactation performance. The individual milk ingredients, milk fat content, and milk protein content in the last DHI before mastitis were also included.

### 4.6. Definition of the Outcome Variables

For statistical analysis, the definition of the levels of severity of mastitis according to the International Dairy Federation was adjusted to a binomial one so that MS 1 and MS 2 were considered non-severe mastitis and MS 3 continued to represent severe mastitis.

### 4.7. Statistical Analysis

For analyzing the dataset, the program SPSS 28.0, IBM, Chicago, IL, USA, was used. Udder quarter with a clinical mastitis case was the statistical unit. Associations between the severity score of occurring clinical mastitis and risk factors (independent variables) were examined with generalized linear mixed models with logit link and binomial response (severe/non-severe (logistic regression)) after pre-screening for variable selection in univariable analysis.

The relation between dependent and independent variables was tested first by appropriate univariable tests. Multicollinearity was checked with Spearman/Kendall’s tau, which indicated a correlation of r > 0.70 with one another. For this reason, no variables were excluded. Then, independent variables associated with the dependent variable at *p* < 0.10 in the univariable test were submitted to generalized linear mixed models.

Using logistic regression procedures, the association between severity and risk factors (independent variables) was examined. Herd, cow within herd, and quarter within cow were considered random effects.

A backward stepwise procedure was used to select the final multivariable regression model. Potential risk factors were excluded if *p* > 0.05.

Meaningful biological interactions between the fixed effects were also used in the final model if significant (*p* < 0.05) and if they did not increase the Akaike Information Criterion (AIC). Non-significant effects or interactions that increased the AIC were not included in the final model. Model fit was evaluated by checking normality of the residuals.

Scaled identity was chosen as the covariance structure because it was assumed that there were no correlations between the elements.

Odds ratios (OR) were calculated to describe the direction of the relationship between dependent and independent variables. OR were determined with 95% confidence intervals (CI 95%) and statistical significance was set at *p* ≤ 0.05.

## 5. Conclusions

In this study, several factors were found to be associated with the severity of clinical expression of mastitis in dairy cattle. Dairy cows treated with corticosteroids for 14 days prior to mastitis were associated with more severe cases of mastitis. It can be concluded that the interaction of coliform pathogens and a high pathogen shedding of these showed associations with severe mastitis. The early lactation stage was an animal-related influencing factor in association with more severe courses of mastitis. As another animal-associated factor, dairy cows predisposed to hypocalcemia were associated with milder clinical expression of mastitis.

## Figures and Tables

**Table 1:** Microbiological results from milk samples from udder quarters (n = 854) with clinical mastitis and their distribution by mastitis severity score.

	Mastitis Severity Score (MS)							
	Mild (MS1)		Moderate (MS2)		Severe (MS3)			
Microbiological findings	n	% ^1^	n	% ^1^	n	% ^1^	Total ^2^ (n)	% ^3^
*Streptococcus* (*Str.*) *uberis*	66	21.6	147	39.9	42	23.3	255	29.9
Coliform pathogens ^4^	40	13.1	74	20.1	76	42.2	190	22.2
No growth	97	31.7	54	14.7	22	12.3	173	20.3
*Non-aureus* *staphylococci* & *Corynebacterium* spp.	50	16.3	23	6.3	5	2.8	78	9.1
Mixed infections	24	7.8	30	8.2	8	4.4	62	7.3
*Staphylococcus* (*S.*) *aureus*	17	5.6	15	4.1	9	5.0	41	4.8
Others ^5^	12	3.9	25	6.7	18	10.0	55	6.4
Total	306 ^2^	100	368 ^2^	100	180 ^2^	100	854 ^6^	100

^1^: proportion of cases of the respective MS. ^2^: number of cases per pathogen group with MS. ^3^: proportion of all clinical cases. ^4^: coliform pathogens: *Escherichia coli*, *Klebsiella* ssp., Enterobacter. ^5^: *Prototheca* spp., *Bacillus* spp., *Enterococcus* spp., yeast, *Pseudomonas* spp., *Streptococcus dysgalactiae*, *Streptococcus agalactiae*, other streptococci, *Trueperella pyogenes*, *Serratia* ssp. ^6^: number of cases with recorded MS.

**Table 2:** Distribution of variables in severity of clinical mastitis cases (n = 854) in herds (n = 65) in northwestern Germany.

	Mild (MS1)		Moderate (MS2)		Severe (MS3)	
Independent Variable	n	% ^1^	n	% ^1^	n	% ^1^
Stage of lactationDIM ^2^ 1–100 DIM ^2^ 101–200 DIM ^2^ > 200	113 78 115	36.9 25.5 37.6	162 102 104	44.0 27.7 28.3	101 53 26	56.1 29.5 14.4
Previous diseases Mastitis Uterine disease Ketosis Hypocalcemia Other diseases	36 19 2 0 7 8	11.8 6.2 0.7 0 2.3 2.6	42 17 1 4 5 15	11.4 4.6 0.3 1.1 1.3 4.1	29 5 8 9 2 5	16.1 2.8 4.4 5.0 1.1 2.8
Corticosteroid treatment No Treatment Treatment	302 4	98.7 1.3	362 6	98.4 1.6	167 13	92.8 7.2
Parity						
First lactation	56	18.3	60	16.3	28	15.6
Second lactation	65	21.2	77	20.9	44	24.4
>Second lactation	185	60.5	231	62.8	108	60.0
Pathogen shedding						
100–1000 cfu ^3^/mL	33	10.8	31	8.4	16	8.9
1100–5000 cfu ^3^/mL	49	16.0	60	16.3	23	12.8
>5000 cfu ^3^/mL	124	40.5	217	59.0	115	63.9
No measurement	100	32.7	60	16.3	26	14.4
Daily milk production (in the last DHI ^4^ test before the case)						
<20 kg ^5^/cow/day	24	7.9	13	3.5	2	1.1
20–40 kg ^5^/cow/day	158	51.8	200	54.4	65	36.2
>40 kg ^5^/cow/day	83	27.2	115	31.3	70	38.9
No measurement	40	13.1	40	10.8	43	23.8
Milk fat content (in the last DHI ^4^ test before the case)						
<4.0%	140	45.7	183	49.7	102	56.6
≥4.0%	166	54.3	185	50.3	78	43.4
Lameness in the last two weeks before mastitis						
No lameness	281	91.8	320	87.0	157	87.2
Lameness	25	8.2	48	13.0	23	12.8
Mastitis in the current lactation						
No mastitis	219	71.6	281	76.4	136	75.6
Mastitis	87	28.4	87	23.6	44	24.4
Body Condition Score (at the time of the case)						
≤2.5	46	15.0	59	16.0	37	20.6
2.75–3.5	194	63.4	239	65.0	107	59.4
≥3.75	66	21.6	70	19.0	36	20.0
Rectal temperature						
≤37.9 °C	89	29.1	49	13.3	8	4.4
38.0–39.4 °C	208	68.0	285	77.5	44	24.5
≥39.5 °C	9	2.9	34	9.2	128	71.1
Localization of the diseased quarter						
Udder quarter rear left	61	19.9	99	26.9	41	22.8
Udder quarter rear right	77	25.2	89	24.2	42	23.4
Udder quarter front left	80	26.1	81	22.0	44	24.4
Udder quarter front right	71	23.2	93	25.3	49	27.2
Several diseased udder quarters	17	5.6	6	1.6	4	2.2
Individual feed intake behavior (at the time of the case)						
Feed intake behavior not maintained	88	28.8	92	25.0	100	55.6
Feed intake behavior maintained	218	71.2	276	75.0	80	44.4
Vaccinations (in the last two weeks before the case)						
Not vaccinated	288	94.1	358	97.3	174	96.7
Vaccinated	18	5.9	10	2.7	6	3.3
Season of the year						
Spring (March–May)	68	22.2	78	21.1	35	19.4
Summer (June–August)	83	27.1	97	26.4	73	40.6
Autumn (September–November)	90	29.4	97	26.4	50	27.8
Winter (December–February)	65	21.3	96	26.1	22	12.2
	306 ^6^	100	368 ^6^	100	180 ^6^	100

^1^: proportion of cases of the respective MS. ^2^: days in milk. ^3^: colony-forming units per milliliter. ^4^: dairy herd improvement. ^5^: kilogram ^6^: number of cases per MS.

**Table 3:** Final generalized linear mixed models with binominal response for severity of clinical mastitis (n = 681; (854 cases–173 no growth cases) in herds (n = 65) in Northwestern Germany.

Effect	ß ^1^	SE ^2^	t Value	OR ^3^	95% CI ^4^ (OR)	*p*-Value
Pathogen group x pathogen shedding ^5^						<0.001
Coliform pathogens × pathogen shedding	0.49	0.10	4.90	1.63	1.34–1.99	<0.001
Others ^6^ × pathogen shedding	0.32	0.15	2.14	1.37	1.03–1.83	0.033
Stage of lactation						0.002
DIM ^7^ 1–100	0.95	0.27	3.46	2.59	1.51–4.44	<0.001
DIM ^7^ 101–200	0.86	0.29	2.93	2.37	1.33–4.23	0.004
DIM ^7^ > 200						Reference
Previous diseases						0.023
Mastitis	−0.70	0.55	−1.28	0.50	0.17–1.45	0.200
Uterine disease	1.23	0.82	1.50	3.43	0.69–17.18	0.133
Ketosis	1.01	0.72	1.41	2.74	0.67–11.20	0.160
Hypocalcemia	−2.29	0.96	−2.37	0.10	0.02–0.67	0.018
Other diseases	−0.60	0.60	−0.99	0.55	0.17–1.79	0.321
No diseases						Reference
Corticosteroid treatment						0.003
No treatment						Reference
Treatment	1.81	0.61	2.95	6.11	1.84–20.33	0.003

^1^: regression coefficient. ^2^: standard error of the mean. ^3^: odds ratio. ^4^: 95% confidence interval for OR. ^5^: A significant effect of the interaction between the pathogen group and shedding intensity (ordinal variable) on the severity of mastitis was found only for combinations of the highest shedding level compared to low and medium shedding. Therefore, for the sake of clarity, only the significant results of all possible combinations are presented here in comparison to the lowest shedding level. ^6^: *Prototheca* spp., *Bacillus* spp., *Enterococcus* spp., yeast, *Pseudomonas* spp., *Streptococcus dysgalactiae*, *Streptococcus agalactiae*, other streptococci, *Trueperella pyogenes*, *Serratia* ssp. ^7^: days in milk.

## Data Availability

Not applicable.

## References

[1] Krömker V., Leimbach S. (2017). Mastitis treatment—Reduction in antibiotic usage in dairy cows. Reprod. Dom. Anim..

[2] International Dairy Federation (IDF) (1999). Suggested Interpretation of Mastitis Terminology.

[3] Schmenger A., Krömker V. (2020). Characterization, cure rates and associated risks of clinical mastitis in Northern Germany. Vet. Sci..

[4] Oliveira L., Holland C., Ruegg P.L. (2013). Characterization of clinical mastitis occurring in cows on 50 large dairy herds in Wisconsin. J. Dairy Sci..

[5] Wenz J.R., Barrington G.M., Garry F.B. (2001). Bacteremia associated with naturally occurring acute coliform mastitis in dairy cows. J. Am. Vet. Med. Assoc..

[6] McDougall S., Agnew K.E., Cursons R., Hou X.X., Compton C.R.W. (2007). Parenteral treatment of clinical mastitis with tylosin base or penethamate hydroiodide in dairy cattle. J. Dairy Sci..

[7] Rees A., Fischer-Tenhagen C., Heuwieser H. (2017). Udder firmness as a possible indicator for clinical mastitis. J. Dairy Sci..

[8] Cheng W.N., Han S.G. (2020). Bovine mastitis: Risk factors, therapeutic strategies, and alternative treatments—A review. Asian-Australas J. Anim. Sci..

[9] Verl. der D. Veterinärmed. G., German Veterinary Association (GVA) (2012). Leitlinien zur Bekämpfung der Mastitis des Rindes als Bestandsproblem. Guidelines for Combating Bovine Mastitis as a Stock Problem.

[10] Brennecke J., Falkenberg U., Wente N., Krömker V. (2021). Are severe mastitis cases in dairy cows associated with bacteremia?. Animals.

[11] International Dairy Federation (IDF) (2011). Suggested Interpretation of Mastitis Terminology.

[12] Burvenich C., Van Merris V., Mehrzad J., Diez-Fraile A., Duchateau L. (2003). Severity of *E. coli* mastitis is mainly determined by cow factors. Vet. Res..

[13] Bradley A.J., Green M.J. (2001). Aetiology of clinical mastitis in six Somerset dairy herd. Vet Record..

[14] Schukken Y.H., Gunther J., Fitzpatrick J., Fontaine M.C., Goetze L., Holst O., Leigh J., Petzl W., Schuberth H.J., Sipka A. (2011). Host-response patterns of intramammary infections in dairy cows. Vet. Immunol. Immunopathol..

[15] Wenz J.R., Barrington G.M., Garry F.B., Ellis R.-P., Magnuson R.J. (2006). *Escherichia coli* isolates serotypes, genotypes, and virulence genes and clinical coliform mastitis severity. J. Dairy Sci..

[16] Bean A., Williamson J., Cursons R.T. (2004). Virulence genes of *Escherichia coli* strains isolated from mastitis milk. J. Vet. Med. B Infect. Dis. Vet. Public Health.

[17] Suojala L., Pohjanvirta T., Simojoki H., Myllyniemi A.L., Pitkälä A., Pelkonen S., Pyörälä S. (2011). Phylogeny, virulence factors and antimicrobial susceptibility of *Escherichia coli* isolated in clinical bovine mastitis. Vet. Microbiol..

[18] Vangroenweghe F., Lamote I., Burvenich C. (2005). Physiology of the periparturient period and its relation to severity of clinical mastitis. Domest. Anim. Endocrinol..

[19] Paape M.J., Mehrzad J., Zhao X., Detilleux J., Burvenich C. (2002). Defense of the bovine mammary gland by polymorphonuclear neutrophil leukocytes. J. Mammary Gland Biol. Neoplasia..

[20] Detilleux J.C. (2002). Genetic factors affecting susceptibility of dairy cows to udder pathogens. Vet. Immunol. Immunopathol..

[21] Kremer W.D.J., Noordhuizen-Stassen E.N., Grommers F.J., Schukken Y.H., Heerings R., Brand A. (1993). Severity of experimental *Escherichia coli* mastitis in ketonemic and nonketonemic dairy cows. J. Dairy Sci..

[22] Mallard B.A., Dekkers J.C., Ireland M.J., Leslie K.E., Sharif S., Vankampen C.L., Wagter L., Wilkie B.N. (1998). Alteration in immune responsiveness during the peripartum period and its ramification on dairy cow and calf health. J. Dairy Sci..

[23] Kimura K., Goff J.P., Kehrli M.E., Reinhardt T.A. (2002). Decreased neutrophil function as a cause of retained placenta in dairy cattle. J. Dairy Sci..

[24] Schukken Y.H., Erb H.N., Smith R.D. (1988). The relationship between mastitis and retained placenta in a commercial population of Holstein dairy cows. Prev. Vet. Med..

[25] Nagasawa Y., Kiku Y., Sugawara K., Yabusaki T., Oono K., Fujii K., Suzuki T., Maehana K., Hayashi T. (2018). The bacterial load in milk is associated with clinical severity in cases of bovine coliform mastitis. J. Vet. Med. Sci..

[26] Sharma N., Singh N.K., Singh O.P., Pandey V., Verma P.K. (2011). Oxidative stress and antioxidant status during transition period in dairy cows. Asian-Australas J. Anim. Sci..

[27] Hogan J.S., Weiss W.P., Smith K.L. (1993). Role of vitamin E and selenium in host defense against mastitis. J. Dairy Sci..

[28] Smith K.L., Hogan J.S., Weiss W.P. (1997). Dietary vitamin E and selenium affect mastitis and milk quality. J. Anim. Sci..

[29] Mehrzad J., Duchateau L., Pyörälä S., Burvenich C. (2002). Blood and milk neutrophil chemiluminescence and viability in primiparous and pluriparous dairy cows during late pregnancy, around parturition and early lactation. J. Dairy Sci..

[30] Vangroenweghe F., Duchateau L., Burvenich C. (2004). Moderate inflammatory reaction during experimental *E. coli* mastitis in primiparous cows. J. Dairy Sci..

[31] Adlam C., Kerry J.B., Edkins S., Ward P.D. (1981). Local and systemic antibody responses in cows following immunization with staphylococcal antigens in the dry period. J. Comp. Pathol..

[32] Cullor J. (1991). The *Escherichia coli* J5 vaccine: Investigating a new tool to combat coliform mastitis. Vet. Med..

[33] Gomes F., Henriques M. (2016). Control of bovine mastitis: Old and recent therapeutic approaches. Curr. Microbiol..

[34] Bradley A.J., Green M.J. (2001). Adaptation of *Escherichia coli* to the bovine mammary gland. J. Clin. Microbiol..

[35] Leelahapongsathon K., Piroon T., Chaisri W., Suriyasathaporn W. (2016). Factors in dry period associated with intramammary infection and subsequent clinical mastitis in early postpartum cows. Asian-Australas. J. Anim. Sci..

[36] Burvenich C., Monfardini E., Mehrzad J., Capuco A.V., Paape M.J. (2004). Role of neutrophil polymorphonuclear leukocytes during bovine coliform mastitis: Physiology or pathology?. Verh K Acad. Geneeskd Belg..

[37] Seffner W., Bergmann A., Wendt K., Bostedt H., Mielke H., Fuchs H.W. (1994). Staphylococcal Infection. Udder and Udder Diseases.

[38] Schulz J., Wendt K., Bostedt H., Mielke H., Fuchs H.W. (1994). Diseases of the Mammary Gland of Cattle. Udder and Udder Diseases.

[39] Roth J.A., Kaeberle M.L. (1982). Effect of glucocorticoids on the bovine immune system. JAVMA.

[40] Marcus E., Kehrli J.R., Jesse P.G. (1989). Periparturient hypocalcemia in cows: Effects on peripheral blood neutrophil and lymphocyte function. J. Dairy Sci..

[41] Bradley A.J., Green M.J. (2001). An investigation of the impact of intramammary antibiotic dry cow therapy on clinical coliform mastitis. J. Dairy Sci..

[42] Olde Riekerink R.G.M., Barkema H.W., Kelton D.F., Scholl D.T. (2008). Incidence rate of clinical mastitis on Canadian dairy farms. J. Dairy Sci..

[43] Samson O., Gaudout N., Schmitt E., Schukken Y.H., Zadoks R. (2016). Use of on-farm data to guide treatment and control auf mastitis caused by *Streptococcus uberis*. J. Dairy Sci..

[44] Persson Y., Nyman A.K.J., Grönlund-Andersson U. (2011). Etiology and antimicrobial susceptibility of udder pathogens from cases of subclinical mastitis in dairy cows in Sweden. Acta Vet. Scand..

[45] Bausewein M., Mansfeld R., Doherr M.G., Harms J., Sorge U.S. (2022). How Good is the Detection of Clinical Mastitis in Automated Milking Systems?.

[46] Klaas I.C., Zadoks R.N. (2018). An update on environmental mastitis: Challenging perceptions. Transbound. Emerg. Dis..

[47] Krömker V., Paduch J.H., Klocke D., Zinke C. (2010). Microbiological investigation of culture negative milk samples of clinical mastitis cows. Milk Sci. Int..

[48] Landeskontrollverband Nordrhein-Westfalen e.V. Jahresbericht 2020. https://www.lkv-nrw.de/fileadmin/redaktion/Publikationen/Jahresberichte/LKV-Jahresbericht_2020.pdf.

[49] Heeschen W., Reichmuth J., Tolle A., Zeidler H. (1969). The preservation of milk samples for bacteriological, cytological, and inhibitory biological examination. Milk Sci. Int..

[50] Verl der D. Veterinärmed. G., German Veterinary Association (GVA) (2009). Leitlinien Entnahme von Milchproben unter antiseptischen Bedingungen und Isolierung und Identifizierung von Mastitiserregern. Guidelines for Antiseptic Milk Sampling and Guidelines to Isolate and Identify Mastitis Pathogens.

[51] Edmonson A.J., Lean I.J., Weaver L.D., Farver T., Webster G. (1989). A Body Condition Scoring chart for Holstein dairy cows. J. Dairy Sci..

[52] Metzner M., Heuwieser W., Klee W. (1993). Die Beurteilung der Körperkondition (Body Condition Scoring) im Herdenmanagement.

[53] National Mastitis Council (1999). Laboratory Handbook on Bovine Mastitis.

[54] Watts J.L., Salmon S.A., Yancey J.R. (1993). Use of modified Rambach agar to differentiate *Streptococcus uberis* from other mastitis streptococci. J. Dairy Sci..

